# Evaluation of analgesic and anti-inflammatory activity of purine-2,6-dione-based TRPA1 antagonists with PDE4/7 inhibitory activity

**DOI:** 10.1007/s43440-022-00397-6

**Published:** 2022-08-05

**Authors:** Małgorzata Zygmunt, Marietta Ślusarczyk, Agnieszka Jankowska, Artur Świerczek, Adrian Bryła, Szczepan Mogilski, Grzegorz Kazek, Jacek Sapa, Elżbieta Wyska, Grażyna Chłoń-Rzepa

**Affiliations:** 1 Department of Pharmacodynamics, Faculty of Pharmacy, Jagiellonian University Medical College, 9 Medyczna str., 30-688 Kraków, Poland; 2 Department of Medicinal Chemistry, Faculty of Pharmacy, Jagiellonian University Medical College, 9 Medyczna str., 30-688 Kraków, Poland; 3 Department of Pharmacokinetics and Physical Pharmacy, Faculty of Pharmacy, Jagiellonian University Medical College, 9 Medyczna str., 30-688 Kraków, Poland

**Keywords:** Purine-2,6-diones, TRPA1 antagonists, PDE4/7 inhibitors, Analgesic activity, Anti-inflammatory activity, Antiarthritic effect

## Abstract

**Background:**

To verify the validity of the proposed pain treatment approach, which is based on concomitant blocking of the Transient Receptor Potential Ankyrin 1 (TRPA1) channel and phosphodiesterases (PDEs) 4B/7A activity, we continued our pharmacological studies on 8-alkoxypurine-2,6-diones selected based on previous in vitro screening.

**Methods:**

Derivatives **17**, **31**, and **36** were pharmacologically evaluated in vivo using the formalin test and oxaliplatin-induced neuropathic pain: the von Frey and the cold plate tests, and in the carrageenan-induced edema model. Compound **36**, which turned out to be the most promising, was further evaluated in the collagen-induced arthritis model. The pharmacokinetic parameters of this compound were also estimated.

**Results:**

All the tested compounds exhibited significant analgesic and anti-inflammatory activities. Compound **36** was additionally characterized by an antiarthritic effect and showed a favorable pharmacokinetic profile in rats.

**Conclusion:**

The compounds evaluated in this study represent a new class of derivatives with analgesic and anti-inflammatory activities that involve TRPA1 antagonism and PDE4/7 inhibition.

**Graphical abstract:**

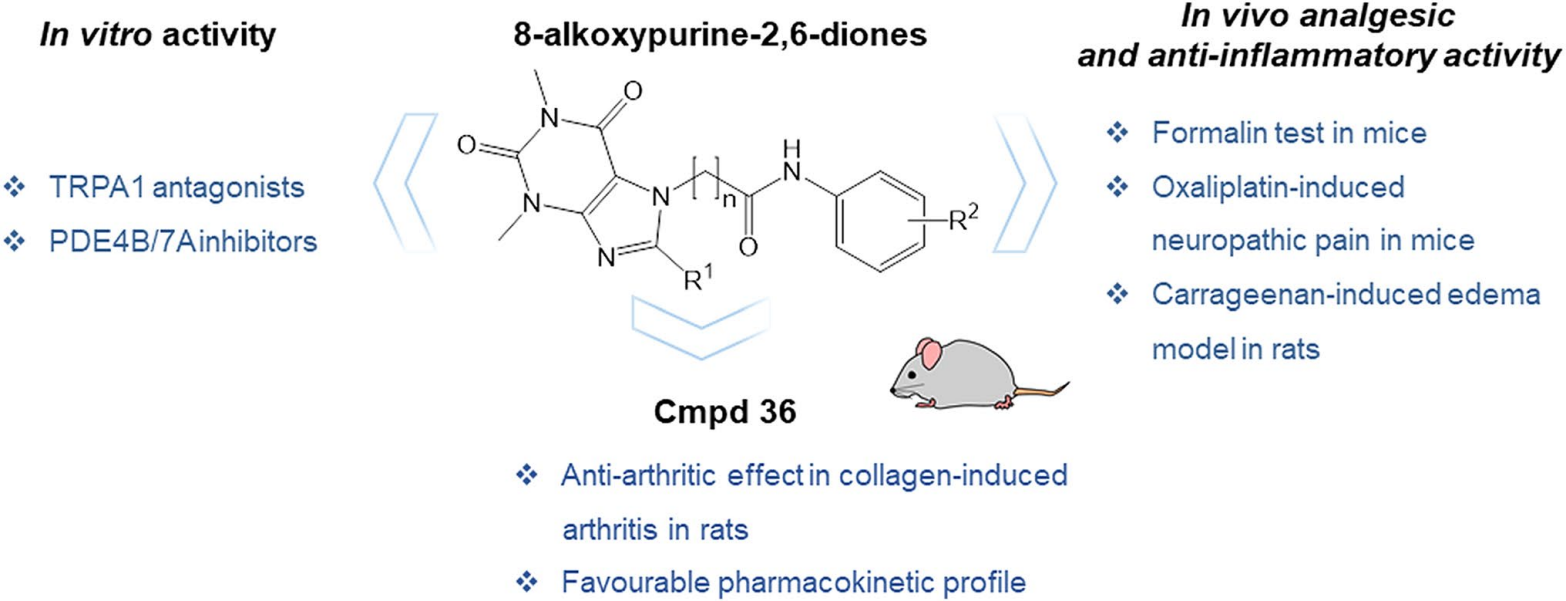

**Supplementary Information:**

The online version contains supplementary material available at 10.1007/s43440-022-00397-6.

## Introduction

Neuropathic pain (NP) is a form of chronic pain caused by a lesion in the somatosensory system, including peripheral fibers and central neurons, or related diseases [1, 2]. Its overall prevalence is estimated at 6.9–10% of the world’s population [3]. NP has a negative impact on the patients’ quality of life, occupational functioning, and general mood, and constitutes a significant burden on health care systems [4]. It is characterized by allodynia that is defined as pain due to stimuli not normally provoking pain and hyperalgesia that is an increased pain response to stimuli that are not normally painful. Numerous factors such as nerve trauma, Herpes virus or HIV infection, cancer or its treatment with platinum-based drugs, or type 2 diabetes can lead to the development of NP [5], and its incidence is expected to rise owing to the aging global population, increased incidence of diabetes mellitus, and improved survival from cancer following chemotherapy. Currently, NP is treated using analgesics, including opioids and adjuvants, such as antidepressants and anticonvulsants which are effective against different pain syndromes. Cancer-related pain is mainly treated with oral opioid analgesics [6]. According to the guidelines of the World Health Organization, when weak opioids such as tramadol or codeine fail to provide satisfactory relief from cancer pain, strong opioids should be incorporated in the treatment plan [7]. Opioids are relatively less effective in alleviating NP than cancer pain, while analgesics despite being effective against some NP types may cause serious side effects leading to non-compliance. As a result, NP remains intractable and its treatment is challenging.

Despite the limitations, advances in the understanding of the pathophysiology of NP are stimulating the development of new diagnostic techniques and personalized interventions, emphasizing the need for a multidisciplinary approach to the management of NP [8].

Nowadays, one of the most relevant targets in the search for novel analgesics for treating NP and inflammatory syndromes is Transient Receptor Potential (TRP) cation channel ligands, especially TRP Ankyrin 1 (TRPA1) channel antagonists [9–11].

TRPA1 is one of the 28 members of the TRP cation channel family. In mammals, it is predominantly expressed in a subset of peripheral sensory neurons, and, thus, play an important role in sensory transduction. It also serves as an irritant sensor for a plethora of electrophilic compounds and as a key mediator of sensory signals with marked effects on cellular function and signaling pathways.

TRPA1 may be activated by different exogenous activators, including cinnamaldehyde, allicin, or allyl isothiocyanate and several endogenous agonists that occur in the course of tissue injury and inflammation, e.g. nitro-oleic acid, formaldehyde, or nonmetabolizable analogues of arachidonic acid. Moreover, evidence indicates that reactive oxygen species, bradykinin, or prostaglandins are TRPA1 agonists. Although many different compounds were identified as TRPA1 antagonists, only few (e.g. purine-2,6-dione-based acetamides including HC-030031) shown efficacy in animal models of inflammatory and acute pain and NP [13].

Inflammatory diseases are also treated by phosphodiesterase (PDE)-targeted therapy, which includes PDE4 and/or PDE7 inhibition. A wide variety of PDE4 inhibitors have been studied in experimental models of inflammation and found to decrease the production of proinflammatory cytokines [13]. An effective strategy for the treatment of inflammatory conditions, including rheumatoid arthritis (RA) and neuroinflammation, is targeting PDE4. PDE7 inhibitors have also been proposed as a potential therapeutic option for the treatment of NP [14].

Some previous studies have analyzed the relationship between structure and analgesic and anti-inflammatory activities of a group of compounds containing a purine-2,6-dione scaffold [15–19]. Among the studied compounds, 1,3-dimethyl-2,6-dioxopurin-7-yl and 8-methoxy-1,3-dimethyl-2,6-dioxopurin-7-yl derivatives of acetic and butanoic acids showed significant analgesic and anti-inflammatory activities in several animal models of pain and inflammation (writhing, formalin, zymosan-induced peritonitis, and carrageenan-induced edema tests) [16, 17, 19]. It was also found that these compounds strongly inhibited PDE, including the PDE4B isoenzyme, compared to the reference inhibitor theophylline [15, 17]. In addition, some of the evaluated compounds significantly inhibited TNF-α production in a rat model of endotoxemia [18, 19]. Furthermore, 8-alkoxy-1,3-dimethyl-2,6-dioxopurin-7-yl-substituted hydrazides exhibited analgesic and anti-inflammatory properties; the latter was probably caused, at least in part, by an increased intracellular level of cAMP.

Recently, our research group proposed the design of a new class of dual PDE4/7 inhibitors, namely butanehydrazides of purine-2,6-dione, and identified several compounds that displayed a strong anti-TNF-α effect in vivo [20]. The amide derivatives of 1,3-dimethyl-2,6-dioxopurin-7-yl-alkylcarboxylic acids were found to show potent anti-inflammatory and analgesic effects with multifunctional TRPA1 antagonistic properties and PDE4/7 inhibitory activity [21]. Among the studied compounds, 4-(8-butoxy-1,3-dimethyl-2,6-dioxo-2,3,6,7-tetrahydro-1H-purin-7-yl)-N-(5-tert-butyl-2-hydroxyphenyl)butanamide (**36**, Table 1) was the most promising and exerted significant antiallodynic properties in animal models of pain and inflammation (formalin test, chemotherapy-induced peripheral neuropathy test in mice, and carrageenan-induced edema test in rats). Moreover, this compound showed a strong anti-TNF-α effect in vivo [21]. These findings indicated that administration of a TRPA1 antagonist with potent PDE4B/7A inhibitory activity may induce an enhanced analgesic effect, at least against certain types of pain, compared to that of pure TRPA1 antagonists. To verify the validity of the proposed pain treatment approach based on the concomitant blocking of the TRPA1 channel and PDE4B/7A activity, we continued our pharmacological studies on purine-2,6-diones [21].

**Table 1:** The structures and TRPA1 channel antagonistic and PDE4B/PDE7A inhibitory activities of evaluated purine-2,6-dione derivatives **17** and **31**


Cmpd	R^1^	R^2^	*n*	hTRPA1% inhibition ± SD		PDE4B	PDE7A
				100 μM	10 μM	IC_50_ [μM]	IC_50_ [μM]
**17**	OCH_3_	4-isopropyl	1	39 ± 0.01	13 ± 0.03	> 200	> 200
**31**	OC_3_H_7_	4*-tert*-butyl	3	52 ± 0.01	5 ± 0.02	21.14	7.76
**36**^a^	OC_4_H_9_	5-*tert*-butyl-2-OH	3	39 ± 0.00	7 ± 0.01	5.43	5.07
HC-030031	H	4- isopropyl	1	43 ± 0.01	13 ± 0.02	> 200	> 200
Rolipram				–		1.1	> 200
BRL-50481				–		175.0	2.1

^a^Data have been published previously [21]

In this study, we evaluated the potential analgesic and anti-inflammatory/antiedematous activities of two selected purine-2,6-dione derivatives: compound **17** (a TRPA1 channel antagonist) and compound **31** (a multifunctional TRPA1 antagonist and a PDE4B/7A inhibitor) (Table 1). The activities of the compounds were examined in animal models of tonic pain and NP (formalin and oxaliplatin-induced NP tests in mice) and inflammation (carrageenan-induced edema test in rats). Compound **36** previously investigated in all above mentioned models was used for comparison. To investigate whether the proposed approach may be useful in the treatment of other diseases associated with inflammation and pain, compound **36** was administered for the first time to rats with collagen-induced arthritis (CIA) which is an established model of human RA. In addition, the pharmacokinetic profile of compound **36** was determined in two strains of rats used in the pharmacological evaluation.

## Materials and methods

### Chemistry

Purine-2,6-dione derivatives **17**, **31**, and **36** evaluated in the study were synthesized according to a previously described multistep procedure [21]. The structures of these compounds are presented in Table 1.

### Assessment of human TRPA1 channel antagonistic activity

To determine the ability of the investigated compounds **17** and **31** to block the TRPA1 channel, a fluorometric calcium imaging assay was carried out using HEK293 cells stably expressing human TRPA1 channel [21]. Briefly, cells were cultured according to the recommendations of the provider (SB Drug Discovery, UK) and subsequently prepared as described previously [21]. Cells grown in 96-well plates were intracellularly loaded with Fluo-4, a Ca^2+^-sensitive fluorescent dye. Then, cells were incubated with a tested compound and monitored for real-time fluorescence changes after the addition of flufenamic acid as a reference agonist. Fluorescence readings (excitation 485 nm, emission 520 nm) were obtained using an Omega POLARStar microplate reader (BMG Labtech, Germany) with a two-channel automatic dispenser. The results were expressed as percentage inhibition of the TRPA1 channel activity achieved with 10 and 100 μM concentrations of the antagonist.

## Pharmacology

### Animals

The in vivo experiments were carried out on male albino Swiss mice (weight 18–26 g) and male Wistar rats (weight 150–180 g) obtained from the Laboratory Animal Facility at the Faculty of Pharmacy, Jagiellonian University Medical College, Kraków, Poland. Female Lewis rats aged 7–9 weeks (weight ~ 180 g) were purchased from Mossakowski Medical Research Center Polish Academy of Sciences, Warsaw, Poland. The animals were housed in facilities under constant temperature and 12:12 light–dark cycle. They were maintained on a standard pellet diet and provided tap water ad libitum*.* The control and experimental groups consisted of 6–8 animals each. In pharmacological studies, the investigated compounds were administered intraperitoneally (ip), whereas in the pharmacokinetic study they were administered both ip and intravenously (iv). The control animals received an equivalent volume of vehicle.

All procedures were conducted in accordance with the guidelines of the International Council on Laboratory Animals Science (ICLAS) and approved by the Local Ethics Committee on Animal Experimentation of the Jagiellonian University in Krakow (agreement no. 47/2014 and 325/2017).

### Formalin test

Before the test, the mice were *ip* administered with the test compound **17** or **31** (20, 10, or 5 mg/kg, as a suspension in methylcellulose) or vehicle (methylcellulose; Avantor Performance Materials, Poland) and allowed to acclimate in Plexiglas observation chambers (20 × 30 × 15 cm) for 60 min. Then, a 5% formalin solution (20 μL) was injected intraplantarly into the right hind paw using a 26-gauge needle, after which the animals were immediately placed individually in glass beakers and observed for half an hour. Time (in sec) spent by animals on licking or biting of the injected paw was recorded in the following intervals: 0–5, 15–20, 20–25, and 25–30 min in each group of animals to evaluate the nociceptive behavior [22]. The ED_50_ values and their confidence limits were calculated as proposed by Litchfield and Wilcoxon [23].

### Oxaliplatin-induced NP—behavioral testing paradigm

Behavioral measures were scored by a trained observer who was blind to experimental conditions. Pain sensitivity threshold was measured prior to oxaliplatin injection (referred to as ‘before oxaliplatin’ measurement), then 3 h after oxaliplatin injection (‘pre-drug’) and 1 h after compounds **17** and **31** administration (‘post-drug’ measurement in acute-phase allodynia). These steps, except for oxaliplatin injection, were repeated 7 days later to assess the antiallodynic effect of **17** and **31** in the late phase of oxaliplatin-induced allodynia [24].

In these experiments, each of the investigated compound was suspended in a methylcellulose and injected *ip* in doses of 1, 5, and 10 mg/kg. The control mice were given a suitable volume of vehicle (methylcellulose). To cause NP, oxaliplatin (Merck, Deutschland) was dissolved in a 5% glucose solution (Polfa Kutno, Poland) and injected ip in a dose of 10 mg/kg 3 h prior to the pain assessment [24].

### Evaluation of mechanical nociceptive threshold (tactile allodynia)—the von Frey test

The electronic von Frey unit (Bioseb, France) with a single flexible filament that applies increasing force (from 0 to 10) against the plantar surface of the animal’s hind paw was used to assess mechanical hypersensitivity (tactile allodynia) in mice. The stimulus was automatically turned off by nocifensive paw withdrawal response, and the mechanical pressure that evoked the response was recorded. To perform measurements, the mice were individually placed in test compartments with a wire mesh bottom and allowed to habituate for an hour. Then, to determine baseline (pre-drug) values of pain sensitivity, the stimulus was applied 3 times alternately in each hind paw of the animals, with an interval of at least 30 s between each measurement. Subsequently, the test compound or vehicle (methylcellulose) were administered as pretreatment. After 60 min, the animals were tested once more to obtain the mean values of postdrug paw withdrawal threshold [24].

### Evaluation of cold nociceptive threshold (cold allodynia)—the cold plate test

The test was performed using the cold plate apparatus (Hot/cold plate, Bioseb, France) at 2 °C. Briefly, the mice were tested to determine baseline latencies to pain reaction. Then, oxaliplatin was injected and latencies to pain reaction (such as biting, lifting, jumping, shaking of hind paws, and movement deficits) were evaluated once more 3 h later (pre-drug latencies). Next, the animals were administered the studied compounds to determine their effect on cold allodynia (postdrug latencies). A cutoff time of 60 s was set up to avoid potential damage in paw tissues. The animals that did not respond within 60 s were taken out of the apparatus and given a score of 60 s.

### Carrageenan-induced edema model

Male Wistar rats were divided into four groups (*n* = 6–8), which included one control group. To induce inflammation, a 1% carrageenan solution in water (100 µL) was injected into the subplantar tissue of the hind paw of rats, as described by C.A. Winter and P. Lence [25, 26]. A plethysmometer (Plethysmometer 7140, Ugo Basile) was used to assess the development of paw edema. Before the injection of the studied compounds, the paw diameters in rats were measured by dividers. The tested compounds **17** and **31** suspended in methylcellulose were given ip in a dose of 20 mg/kg, 60 min before carrageenan administration. Our previous studies revealed that compounds with similar structures to the investigated compounds have shown no activity at a lower dose in this test [21]. The control group was administered ip with methylcellulose which had no effect on edema (data not shown). Ketoprofen was employed as a positive control. The paw diameters were evaluated at 1, 2, and 3 h postdosing. The percentages of edema and edema inhibition were calculated according to the below formulas:

1 $${\text{Edema}} \left( \% \right) = \left( {N^{\prime}\, \times \,100} \right)/N$$

2 $${\text{Edema }}\,{\text{inhibition }}\left( \% \right) = \left( {N - N^{\prime}\, \times \,100} \right)/N$$

where,N—paw diameters assessed 1, 2, and 3 h following carrageenan administration in comparison to the control group paw diameters at the beginning of the experiment.N’—paw diameters assessed 1, 2, and 3 h following carrageenan administration in comparison to the tested group paw diameters at the beginning of the experiment.

### Analgesic activity test

This method for measuring analgesic activity is based on the method described by Randall and Selitto [27]. To induce inflammation, 0.1 mL of a carrageenan solution in saline is injected subcutaneously into the plantar surface of the left hind paw of the rat. Three hours later, pressure is applied through a tip to the plantar surface of the rat’s foot at a constant rate (expressed in g) by an analgesymeter type Ugo Basile, to the point at which the animal struggles, squeals or attempts to bite. The percentage increase in pain threshold is calculated, by subtracting the applied force of the vehicle control from the applied force in the drug group, which is divided by the applied force of the vehicle control to give the percentage of increase in pain threshold of the drug group. Percent of analgesia was calculated according to the following formula: % analgesia = (100 × B)/A–100. Where: A = mean pressure (in g) in the control group; B = mean pressure (in g) in the test group. Mean pain threshold was calculated for treated and control groups as the percent change in relation to the control.

### Collagen-induced arthritis (CIA) model

Fifteen female Lewis rats were acclimatized for 1 week prior to the experiment. The animals were housed in the Laboratory Animal Facility at the Faculty of Pharmacy, Jagiellonian University Medical College, under constant temperature (21 °C), humidity (72%), and 12:12 light–dark cycle. They had free access to rat chow and tap water throughout the study. Arthritis was induced following the instructions of Chondrex, Inc. (Redmond, WA). Briefly, porcine collagen type II (2 mg/mL) in 0.05 M acetic acid was emulsified with incomplete Freund’s adjuvant (Sigma-Aldrich, St. Louis, MO). After the evaluation of paw edema induction on day 20, 12 CIA rats that presented with at least 50% size increase in one or two paws were selected and randomly assigned to two groups: the control group receiving vehicle and the study group administered *ip* once daily with 50 mg/kg compound of **36** dissolved in 10% dimethyl sulfoxide in normal saline. The dosing started from day 20 postinduction. Edema was assessed by the size of the hind paws [28]. CIA was also evaluated on a qualitative 4-point clinical scale, in which 0 indicated normal limb, 1 indicated mild but definite redness and swelling of the ankle or wrist, or apparent redness and swelling limited to individual digits, regardless of the number of affected digits, 2 indicated moderate redness and swelling of the ankle or wrist, 3 indicated severe redness and swelling of the entire paw including digits, and 4 indicated maximally inflamed limb involving multiple joints.

### Statistical analysis

Statistical analysis was performed using GraphPad Prism 5.0 (GraphPad Software LLC) and Statistica v. 13 (TIBCO Software Inc., Palo Alto, CA, USA) software. The normality of data distribution and the homogeneity of variance was checked using a Shapiro–Wilk’s test and Levene’s test, respectively. One-way ANOVA, followed by Dunnett’s post hoc test or Mann–Whitney *U* test was used, where applicable. The differences between means were considered statistically significant at *p* < 0.05. The data are expressed as means ± standard error of the mean.

### Pharmacokinetic study

The pharmacokinetic profile of compound **36** was assessed by administering it iv and ip to cannulated male Wistar or female Lewis rats. Three days before the experiment, the rats were implanted with catheters (SAI Infusion Technologies, USA) into the jugular vein under anesthesia using ketamine and xylazine. After the administration of the compound, blood samples were collected at 15, 30, 60, 90, and 120 min from rats into heparinized tubes and subsequently centrifuged at 3000×*g* for 10 min. Then, plasma samples were harvested and stored at − 80 °C until analysis.

### Analytical method

The level of compound **36** in rat plasma was determined using the previously described high-performance liquid chromatography (HPLC) method with UV detection [21]. Briefly, 10 μL of 10 mg L^–1^ N-(4-(tert-butyl)phenyl)-4-(8-((furan-2-ylmethyl)amino)-1,3-dimethyl-2,6-dioxo-1,2,3,6-tetrahydro-7*H*-purin-7-yl)butanamide methanol solution used as an internal standard (IS) and 20 μL of 1 M HCl solution were added to polypropylene snap-cap tubes containing 100 μL of the investigated plasma or blank plasma spiked with a standard solution of compound **36**. Subsequently, each sample was treated with 1 mL of ethyl acetate and extracted for 20 min on a VXR Vibrax shaker (IKA, Germany). Then, the samples were centrifuged (10,000×*g*, 5 min) on an EBA 12 R centrifuge (Hettich, Germany), and ethyl acetate was collected and transferred to clean tubes. Organic layers were evaporated under a mild stream of nitrogen at 40 °C in a water bath, and the resulting residues were dissolved in 100 μL of the mobile phase. The HPLC system (LaChrom Elite, Merck-Hitachi, Germany) used for the analysis was equipped with an L-2420 UV–Vis detector, L-2200 autosampler, and L-2130 pump. Data acquisition and integration was performed using EZChrome Elite v. 3.2 (Merck-Hitachi, Germany) software. The analysis was performed at ambient temperature on a LiChrospher 100 RP-18 column (250 × 4 mm) protected with a LiChroCART precolumn (4 × 4 mm; Merck, Germany). The mobile phase used for the analysis was composed of acetonitrile and 20 mM KH_2_PO_4_ water solution (pH 4.5) at a ratio of 55:45 (v/v). The flow rate was set to 1 mL min^−1^, and analytical wavelength to 209 nm. The retention times in these conditions for the IS and compound **36** were 13 and 18 min, respectively. The assay was reproducible with low intra- and interday variation (coefficient of variation < 10%). The calibration curves were linear at a range of 0.020–10 mg L^–1^.

### Pharmacokinetic data analysis

The values of the pharmacokinetic parameters were determined by nonlinear regression using ADAPT 5 software (BMSR, Los Angeles, CA). A one-compartment model was simultaneously fitted to both iv and ip concentration–time profiles of compound **36** determined in each rat strain to obtain two sets of pharmacokinetic parameters. The absolute bioavailability (F_a_) of the compound following *ip* administration was calculated according to the following equation:

3 $${F}_{a}=\frac{{\mathrm{AUC}}_{\mathrm{i}.\mathrm{p}.}\bullet {\mathrm{D}}_{\mathrm{i}.\mathrm{v}.}}{{\mathrm{AUC}}_{\mathrm{i}.\mathrm{v}.}\bullet {\mathrm{D}}_{\mathrm{i}.\mathrm{p}}}$$

where AUC_ip_ and AUC_iv_ are the areas under concentration–time profiles calculated from the time of ip and iv dosing to the last time point measured using the trapezoidal rule and D_ip_ and D_iv_ are ip and iv doses administered to rats, respectively.

## Results

To evaluate human TRPA1 channel antagonistic properties of compounds **17** and **31**, kinetic fluorescent determination of calcium influx was performed in human TRPA1-transfected HEK-293 cells. The results were expressed as percentage inhibition of the TRPA1 channel activity achieved with 100 and 10 μM concentrations of the antagonists.

The data presented in Table 1 indicate that among the tested 8-alkoxypurine-2,6-diones, compound **17** was characterized with a similar IC_50_ value to that of HC-030031, a reference TRPA1 channel antagonist [11, 29]. Previous studies revealed that compound **36** displayed dual PDE4B/7A inhibitory activity and was identified as the most active PDE4B/7A inhibitor (monodigital IC_50_ values were in the same range as rolipram and BRL-50481 which are selective PDE4 and PDE7 inhibitors, respectively) [21]. The inhibitory activity of **31** in comparison to **36** was slightly weaker.

Further, the antinociceptive and anti-inflammatory activities of compounds **17** and **31** were tested in animal models of tonic pain and NP (formalin test and oxaliplatin-induced NP test in mice, respectively) and inflammation (carrageenan-induced edema test in rats) in comparison with compound **36**. Accordingly, the previously published data sets for **36** [21] are included in the current study for comparison. This was in line with the recommendation of the Ethics Committees for Animal Experimentation which suggested using the historical controls in experiments with animals. To assess whether simultaneous blocking of the TRPA1 channel and PDE4/7 may be beneficial useful in the treatment of other inflammatory disorders, compound **36** was tested in rats with CIA.

### Antinociceptive activity in the formalin test

The formalin test carried out in mice with chronic pain induced by the administration of 5% formalin solution revealed that compounds **17** and **31** showed antinociceptive activity (Table 2). The injection of formalin into the dorsal surface of the hind paw induces a biphasic nociceptive behavioral response in mice, which includes licking, biting, flinching, or lifting of the injected paw. The acute (neurogenic) nociceptive phase lasts for 5 min, and a little activity is observed during the next 10 min. The first (early) phase of the test involves the stimulation of nociceptors and the development of neurogenic inflammation, while the second (late) phase begins after 15–30 min of formalin injection and involves peripheral inflammation and central sensitization of pain. Since the second phase reflects the activation of inflammatory processes, the compounds active in this phase of the experiment also exhibit an anti-inflammatory effect.

**Table 2:** The influence of the investigated compounds **17** and **31** on the duration of pain reaction in the formalin test in mice

Cmpd	Dose [mg/kg]	Time [s] spent on licking or biting the injected paw ± SEM		ED_50_ [mg/kg]		Effect [%]	Effect [%]
		Phase I	Phase II	Phase I	Phase II	Phase I	Phase II
Control	–	75.5 ± 6.9	143.60	–	–	–	–
**17**	20	67.2 ± 13.2	36.3 ± 11.6^c^	–	6.7	10.9	74.7
	10	63.3 ± 14.6	68.3 ± 18.3^a^			16.1	52.4
	5	82.3 ± 8.5	76.3 ± 12.1			0	46.9
		F_3,20_ = 12.36	F_3,20 _ = 468.3 *p* = 0.0003 *p* = 0.0316				
Control	–	34.6 ± 6.8	105.3 ± 11.0	–	–	–	–
**31**	20 10	19.2 ± 5.8 22.8 ± 3.2	34.5 ± 14.5^a^ 57.6 ± 20.3	–	10.9	44.5 34.1	67.2 45.3
	5	4.8 ± 7.9	73.2 ± 17.1			0	30.5
		F_3,20_ = 36.29	F_3,20_ = 42.48 *p* = 0.0323				
Control	–	33.0 ± 1.4	83.6 ± 13.0	–		–	–
36*	10	21.3 ± 3.8^a^	0.017 ± 0.017^c^			35.4	99.9
	5	25.2 ± 4.7	49.2 ± 10.1	–	3.4	23.6	41.1
	2.5	32.0 ± 7.4	47.7 ± 4.7^a^			0.03	42.9
		F_3,20_ = 2.965 *p* = 0.0398	F_3,20_ = 78.94 *p* = 0.0004 *p* = 0.0398				
Control	–	97.0 ± 6.9	158.3 ± 12.3			–	–
HC-030031*	100	71.0 ± 4.1	13.9 ± 1.3^c^			26.8	91.2
	40	73.8 ± 5.3	76.5 ± 9.0^a^	–	33.8	23.9	51.7
	20	82.8 ± 4.8	111.3 ± 12.2			14.6	29.7
		F_3,20_ = 5.151	F_3,20_ = 138.9 *p* = 0.0362 *p* = 0.0001				

Data are presented as the means ± SEM. *N* = 6–8. One-way ANOVA, followed by Dunnett’s post hoc comparison:

^a^*p* = 0.0316, ^c^*p* = 0.0003 vs. control (**17**); ^a^*p* = 0.0323 vs. control (**31**); ^a^*p* = 0.0398, ^c^*p* = 0.0004 vs. control (**36**); ^a^*p* = 0.0362, ^c^*p* = 0.0001 vs. control (**HC-030031**)

*Data have been published previously [21]

The tested compounds shortened the time spent by mice licking or biting the right hind paw in response to the irritating chemical stimulus. The data presented in Table 2 indicate that at the first (neurogenic) phase of the test (0–5 min) only compound **36** showed a statistically significant antinociceptive activity, while the other two compounds were active in the second (inflammatory) phase of the test (15–30 min), however, at higher doses than compound **36**. A comparison of the ED_50_ values (Table 2) highlighted that all the tested 8-alkoxypurine-2,6-diones showed a stronger antinociceptive and anti-inflammatory effect than that observed with the administration of HC-030031, a model TRPA1 antagonist. Compound **36** displayed the most potent antinociceptive activity with a tenfold higher effect than that of HC-030031. Comparing the ED_50_ values calculated for compounds **17** and **31** with that of HC-030031, it was found that their antinociceptive activity was five- and threefold higher, respectively (Table 2).

### Oxaliplatin-induced NP test

It is well known that TRPA1 channels may be activated by substances sensitive to glutathione, such as reactive oxygen species produced as a result of tissue exposure to platinum-based compounds. This mechanism and the previously discovered pathway of TRPA1 activation, are the major contributor to the transition of mechanical and cold hypersensitivity from acute to chronic pain. In this study, we investigated the effects of the studied compounds in a mouse model of oxaliplatin chemotherapy-induced peripheral neuropathy (the von Frey and the cold plate tests).

### Influence on tactile allodynia (the von Frey test)

The von Frey test revealed that compound **17** showed significant antiallodynic properties in both acute and late phases of oxaliplatin-induced NP (Table 3). Post hoc analyses showed that the compound was effective at a dose of 5 mg/kg in the early phase (*p* < 0.001) and at 10 mg/kg in both early and late phases (*p* < 0.001 and *p* < 0.01, respectively). In turn, compound **31** displayed antiallodynic properties in the late phase at all three doses. On the other hand, compound **36** displayed significant antiallodynic properties in the early phase only at a dose of 10 mg/kg (*p* < 0.001 vs. pre-drug paw withdrawal threshold) [21]. Taken together, in the von Frey test, compound **31** showed the most pronounced antiallodynic activity by attenuating tactile allodynia in both phases of oxaliplatin-induced neuropathy and was active even at the lowest dose in the late phase.

**Table 3:** The influence of the investigated compounds **17** and **31** on the paw withdrawal thresholds in the von Frey test

Cmpd	Dose [mg/kg]	Paw withdrawal threshold (g)			
		Early phase (3th hour)		Late phase (7th day)	
		Pre-drug	Post-drug	Pre-drug	Post-drug
Control		2.98 ± 0.06			
	10	1.73 ± 0.04	2.20 ± 0.08^c^	1.91 ± 0.04	2.15 ± 0.05^b^
**17**	5	1.79 ± 0.04	2.17 ± 0.12^b^	1.96 ± 0.08	2.11 ± 0.05
	1	1.71 ± 0.06	1.81 ± 0.07 F_14,75_ = 21.90 *p* = 0.0096 *p* = 0.0001	1.78 ± 0.05	1.92 ± 0.08 *p* = 0.0094
Control		3.04 ± 0.07			
	10	1.78 ± 0.05	2.15 ± 0.06^a^	2.09 ± 0.05	2.38 ± 0.02^b^
**31**	5	1.82 ± 0.03	2.11 ± 0.05^b^	1.91 ± 0.05	2.14 ± 0.06^a^
	1	1.80 ± 0.04	1.97 ± 0.05 F_14,74_ = 17.92 *p* = 0.0142 *p* = 0.0087	1.79 ± 0.04	1.87 ± 0.04^a^ *p* = 0.0438 *p* = 0.0022
Control		2.61 ± 0.16			
	10	1.72 ± 0.07	2.88 ± 0.15^c^	1.70 ± 0.10	2.60 ± 0.00
36*	5	1.80 ± 0.05	2.16 ± 0.15	1.85 ± 0.06	2.40 ± 0.09
	1	1.78 ± 0.06	1.94 ± 0.10 F_14,75_ = 18.07 *p* = 0.00046	1.83 ± 0.03	1.90 ± 0.10
Control					
	10	1.83 ± 0.08	1.90 ± 0.04		
**HC-030031**	5	1.86 ± 0.07	1.95 ± 0.06		
	1	1.71 ± 0.06	1.84 ± 0.07 F_14,75_ = 24.19		
Control		3.00 ± 0.08			
	10	1.89 ± 0.08	3.76 ± 0.19^c^	1.86 ± 0.07	3.96 ± 0.05^c^
Pregabalin^*^	5	1.85 ± 0.06	2.98 ± 0.06^c^	1.68 ± 0.05	2.89 ± 0.09^c^
	1	1.80 ± 0.09	1.91 ± 0.08 F_14,75_ = 23.86 *p* = 0.0001	1.75 ± 0.05	2.05 ± 0.09 *p* = 0.0003

Data are presented as the means ± SEM. *N* = 6–8. One-way ANOVA, followed by Dunnett’s post hoc ^b^*p* = 0.0096, ^c^*p* = 0.0001 vs. pre-drug paw withdrawal threshold, early phase; ^b^*p* = 0.0094 vs. pre-drug paw withdrawal threshold, late phase (**17**) ^a^*p* = 0.0142, ^b^*p* = 0.0087 vs. pre-drug paw withdrawal threshold, early phase; ^a^*p* = 0.0438, ^b^*p* = 0.0022 vs. pre-drug paw withdrawal threshold; late phase (**31**); ^c^*p* = 0.00046 vs. pre-drug paw withdrawal threshold, early phase (**36**); ^c^*p* = 0.0001 vs. pre-drug paw withdrawal threshold, early phase; significance: ^c^*p* = 0.0003 vs. pre-drug paw withdrawal threshold; late phase (pregabalin)

*Data have been published previously [21]

Pregabalin displayed statistically significant antiallodynic properties in both phases of oxaliplatin-induced peripheral neuropathy attenuating tactile allodynia at both doses, (i.e. 5 and 10 mg/kg; significant at *p* < 0.001). Its effect was slightly stronger compared to those of compounds **17** and **31** [21].

In this test, TRPA1 antagonist **HC-030031** demonstrated no statistically significant antiallodynic properties in both phases of oxaliplatin-induced peripheral neuropathy, administered at doses 1.5 and 10 mg/kg. In turn, it has been demonstrated previously that **HC-030031** (at dose 100 mg/kg) showed significant antiallodynic properties both in the acute phase and in the late phase of oxaliplatin-induced neuropathic pain in mice [21, 24].

### Influence on cold allodynia (the cold plate test)

As shown in Table 4, in the cold plate test, compounds **17** and **31** exhibited an antiallodynic effect in oxaliplatin-treated mice. Post hoc analyses showed that compound **17** effectively attenuated cold allodynia at a dose of 10 mg/kg in both early and late phases (*p* < 0.01 and *p* < 0.05, respectively). Compound **31** also displayed antiallodynic properties at a dose of 10 mg/kg (*p* < 0.05) in the both phases of cold allodynia (*p* < 0.05). Compound **36** showed no effect on the cold nociceptive threshold of oxaliplatin-treated mice, in the early or late phase, at any of the doses used [21].

**Table 4:** The influence of the investigated compounds **17** and **31** on the threshold of pain sensitivity in the cold plate test

Cmpd	Dose [mg/kg]	Response latency [s]			
		Early phase (3th hour)		Late phase (7th day)	
		Pre-drug	Post-drug	Pre-drug	Post-drug
Control		38.68 ± 5.65			
	10	24.86 ± 2.39	43.26 ± 4.80^b^	23.41 ± 3.02	37.40 ± 4.44^a^
**17**	5	22.62 ± 3.82	24.00 ± 3.24	17.63 ± 2.08	22.21 ± 3.84
	1	11.59 ± 2.70	19.43 ± 4.23 F_14,75_ = 38.21 *p* = 0.0022	11.44 ± 2.90	13.52 ± 4.14 *p* = 0.0186
Control		41.36 ± 4.46			
	10	21.34 ± 3.03	32.23 ± 4.22^a^	17.59 ± 2.18	28.13 ± 4.37^a^
**31**	5	31.58 ± 5.62	34.83 ± 6.27	18.96 ± 1.98	29.35 ± 3.14
	1	19.24 ± 3.95	21.63 ± 3.98 F_14,75_ = 30.06 *p* = 0.0323	21.38 ± 3.03	24.59 ± 3.13 *p* = 0.0169
Control		60.00 ± 0.00			
	10	11.22 ± 2.11	10.94 ± 4.47	12.75 ± 7.45	16.30 ± 9.60
36*	5	33.20 ± 3.30	38.93 ± 8.93	36.05 ± 23.95	11.25 ± 6.95
	1	7.88 ± 2.05	17.80 ± 2.68 F_14,75_ = 28.16	23.77 ± 5.06	11.50 ± 6.84
Control		58.00 ± 2.25			
	10	22.43 ± 3.54	27.78 ± 2.52	19.38 ± 3.15	25.41 ± 1.96
**HC-030031**	5	31.25 ± 2.18	33.51 ± 5.83	21.89 ± 4.52	25.87 ± 4.29
	1	24.86 ± 3.56	27.02 ± 4.51 F_14,75_ = 24.31	23.78 ± 2.98	26.51 ± 3.54
Control		58.00 ± 2.25			
	10	19.34 ± 2.51	26.27 ± 3.25	18.68 ± 4.37	21.43 ± 1.34
Pregabalin*	5	23.64 ± 1.98	25.48 ± 4.17	21.37 ± 3.04	25.39 ± 3.26
	1	25.63 ± 3.29	27.28 ± 3.86 F_14,75_ = 32.07	23.45 ± 4.21	25.07 ± 4.15

Data are presented as the means ± SEM. *N* = 6–8. One-way ANOVA, followed by Dunnett’s post hoc comparison:

^b^*p* = 0.0022 vs. pre-drug paw withdrawal threshold, early phase

^a^*p* = 0.0186 vs. pre-drug paw withdrawal threshold, late phase (**17**)

^a^*p* = 0.0323 vs. pre-drug paw withdrawal threshold; early phase

^a^*p* = 0.0169 vs. pre-drug paw withdrawal threshold, late phase (**31**)

*Data have been published previously [21]

In the cold plate test, post hoc analysis revealed that pregabalin did not attenuate cold allodynia, either in the early, or in the late phase of neuropathy caused by oxaliplatin, at any dose used. This is in line with our earlier studies which also demonstrated that lower doses of pregabalin (i.e. doses below 10 mg/kg) were not able to attenuate cold allodynia in oxaliplatin-treated mice [21, 30]. In this test, **HC-030031** did not attenuate cold allodynia, either in the early, or in the late phase of neuropathy caused by oxaliplatin, at any dose used. In turn, it has been previously reported that TRPA1 antagonist HC-030031 (100 mg/kg) attenuated oxaliplatin-induced acute cold hypersensitivity in mice [24]. This compound also effectively reduced oxaliplatin-evoked cold hypersensitivity in rats [31].

### Anti-inflammatory (antiedematous) effect in the carrageenan-induced edema model

In the carrageenan test performed to evaluate the anti-inflammatory (antiedematous) activity of compounds **17** and **31**, the polysaccharide carrageenan was injected into the hind paw of rats to induce long-lasting edema.

The test revealed that compound **31** significantly decreased the volume of edema induced by carrageenan in the hind paw of rats (Table 5). The decrease in paw edema by 62.0 and 48.3%, was evident at 1 and 2 h of the experiment, respectively. Similarly, compound **36** showed anti-inflammatory (antiedematous) activity at 1 and 2 h of the experiment and decreased edema formation by 48.0 and 79.8%, respectively [21]. Compound **17** did not show statistically significant antiedematous activity. Ketoprofen, which was used as a second reference compound, inhibited edema formation by 59.5, 64.3, and 67.7% in three consecutive hours of the experiment, respectively (Table 5); however, this effect was statistically significant only at 2 and 3 h after dosing. HC-030031 administered at a dose of 100 mg/kg statistically significantly inhibited edema development at 2 and 3 h of the experiment by 48.2 and 48.3%, respectively [21]. All the compounds examined in the carrageenan-induced edema model, showed significant analgesic activity. Compound **36** showed the strongest analgesic effect (63%) in comparison to that observed for **17** (25,4%) and **31** (43.1%), (Table 5).

**Table 5:** Anti-inflammatory (antiedematous) effect of compounds **17** and **31** administered *ip* at a dose of 20 mg/kg in the carrageenan-induced paw edema test in rats

Cmpd	Change in edema volume [mL]			Analgesic activity [%]
	1 h	2 h	3 h	
Control	0.79 ± 0.04	1.12 ± 0.13	1.33 ± 0.13	
**17**	0.56 ± 0.14	0.73 ± 0.10 F_1,10_ = 5.445	1.11 ± 0.21	25.4
Control	0.42 ± 0.07	0.89 ± 0.11	1.25 ± 0.06	
**31**	0.16 ± 0.05^a^ *p* = 0.0438	0.46 ± 0.08^a^ F_1,10_ = 2.585 *p* = 0.0438	1.68 ± 0.06	43.1
control	0.42 ± 0.07	0.89 ± 0.11	1.25 ± 0.06	
36*	0.22 ± 0.06^a^ *p* = 0.0398	0.18 ± 0.01^b^ F_1,10_ = 5.994 *p* = 0.0082	0.83 ± 0.9	63.4
Control	0.79 ± 0.03	1.12 ± 0.13	1.33 ± 0.13	
Ketoprofen*	0.32 ± 0.02	0.40 ± 0.03^a^ F_1,10_ = 3.446 *p* = 0.0316	0.43 ± 0.03^b^ *p* = 0.0096	107.8

Data are presented as the means ± SEM. *N* = 6–8. One-way ANOVA, followed by Dunnett’s post hoc comparison:

^a^*p* = 0.0438 vs. control (**31**)

^a^*p* = 0.0398

^b^*p* = 0.0082 vs. control (**36**)

^a^*p* = 0.0316

^b^*p* = 0.0096 vs. control (ketoprofen)

*Data have been published previously [21]

**Fig. 1:**
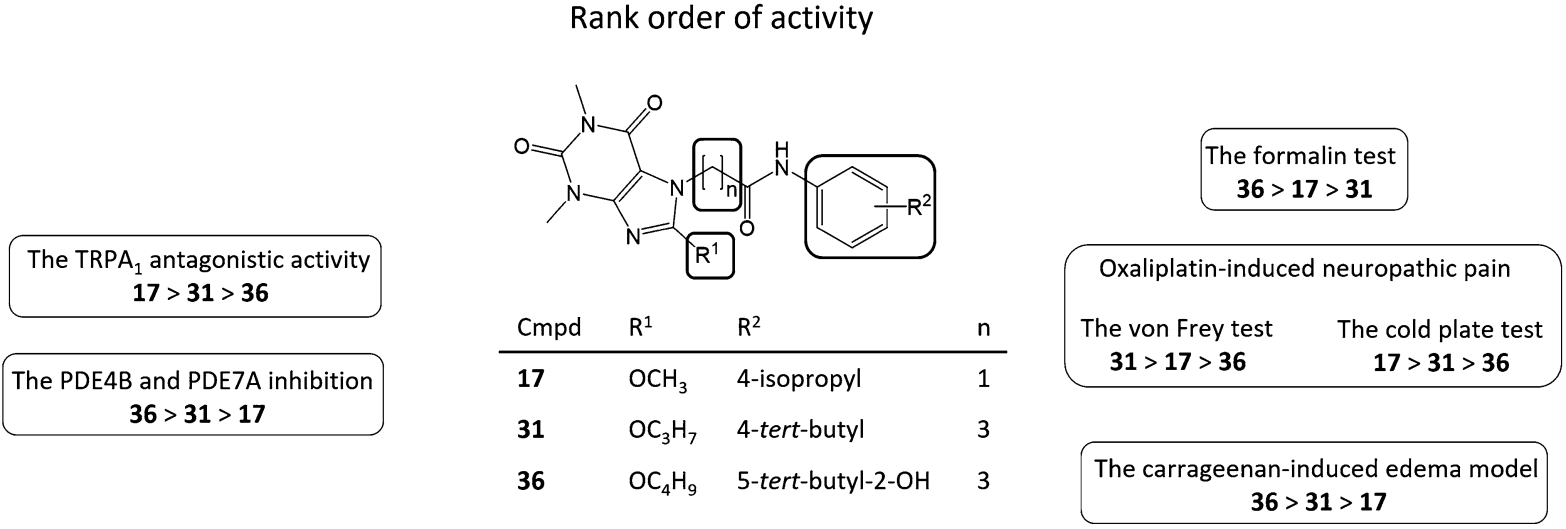
Summary of SAR studies of the tested compounds for analgesic and anti-inflammatory activity

The structure–activity relationship (SAR) study of tested compounds **17** and **31** and reference compound **36** determined the preferred structural features for analgesic and anti-inflammatory activity (Fig. 1). Compounds with propylene aliphatic chain (**31** and **36**) as PDE4B/7A inhibitors exerted the most significant anti-inflammatory activity in the carrageenan-induced edema model.. On the other hand the methylene linker (**17)** was more preferable for oxaliplatin-induced neuropathic pain and probable it was connected with the most potent TRPA1antagonistic activity of **17.**

### Anti-inflammatory activity in the CIA model

Compound **36**, a potent dual PDE4/7 inhibitor and TRPA1 antagonist, exerted strong anti-TNF-α activity in vivo in lipopolysaccharide (LPS)-induced endotoxemia, which was manifested by a significant reduction in the maximum plasma concentration of TNF-α by 90.2% [21]. On the other hand, the anti-TNF-α potential was not observed for compounds with only TRPA1 antagonistic properties [21]. Taking this into account and the fact that TNF-α is a key cytokine in the course of RA development, compound **36** was further evaluated in the current study in rats with CIA a well-recognized model of human RA.

**Fig. 2:**
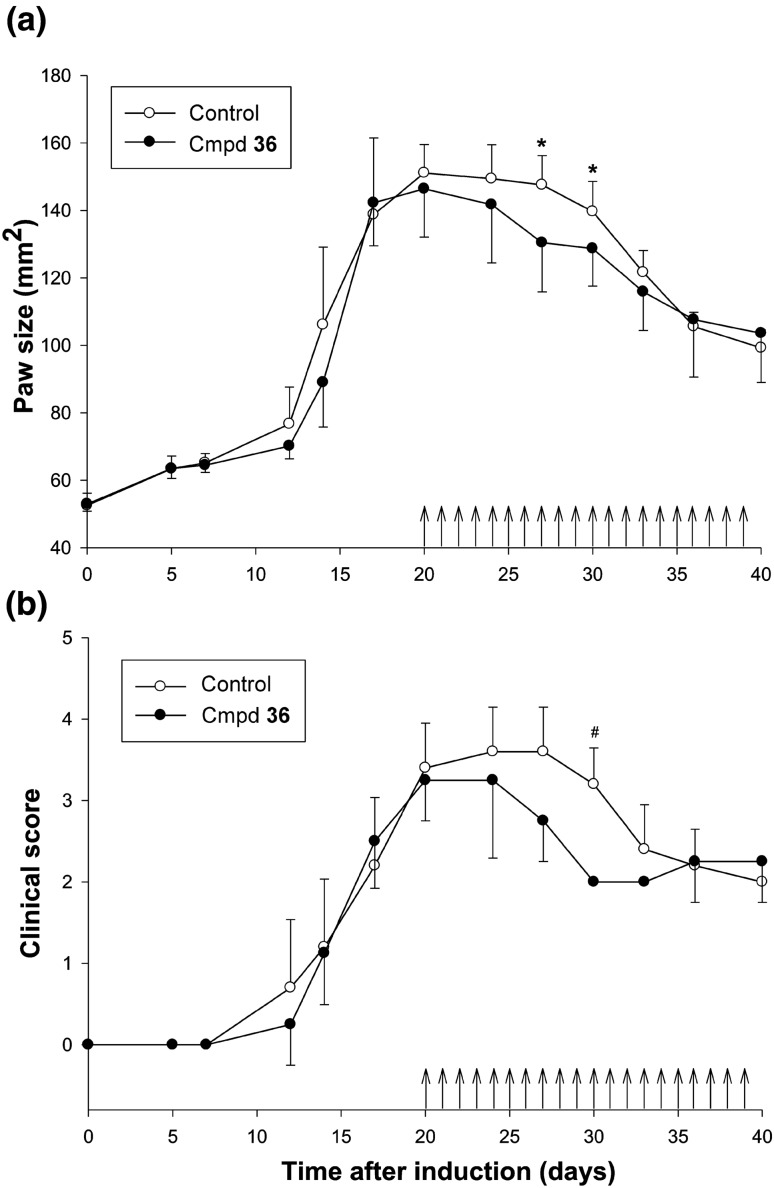
Pharmacodynamic profiles of compound **36** in the control group and rats with CIA; paw size as a function of time (**a**) and changes of clinical score over time (**b**). The compound dosing started from day 20 post-induction (*N* = 6, ^a^*p* < 0.03, Mann–Whitney *U* test).*p = 0.02, *U* = 3, *N*_1_ = 6, *N*_2_ = 6 (*N*_1_ and *N*_2_ are sample sizes for groups). #*p* = 0.005, *U* = 0, *N*_1_ = 6, *N*_2_ = 6

The data presented in Fig. 2 indicate that after the first 7 days of disease induction, gradual paw growth was noted, but with no apparent disease symptoms.

On day 12 of the experiment, there was a rapid increase in the clinical score value and paw area in the group treated with compound **36** and the control group. The peak clinical score values were reached on day 24 and paw size values on day 20. On subsequent days, the clinical score and paw area decreased; however, in the group treated with compound **36**, the initial decrease in values was sharper than that in the control group receiving vehicle. Compared to the control group, the group treated with compound **36** had significantly smaller paw areas on days 27 and 30 after the first immunization and significantly lower clinical score value on day 30. The paw areas in the compound **36**-treated group were reduced by 11.6 and 7.9% on days 27 and 30, respectively. At most of the remaining measurement points, differences were observed between the two groups in paw swelling and clinical score; however, they did not reach statistical significance (*p* > 0.05), probably due to large data variability.

### Pharmacokinetic study

**Fig. 3:**
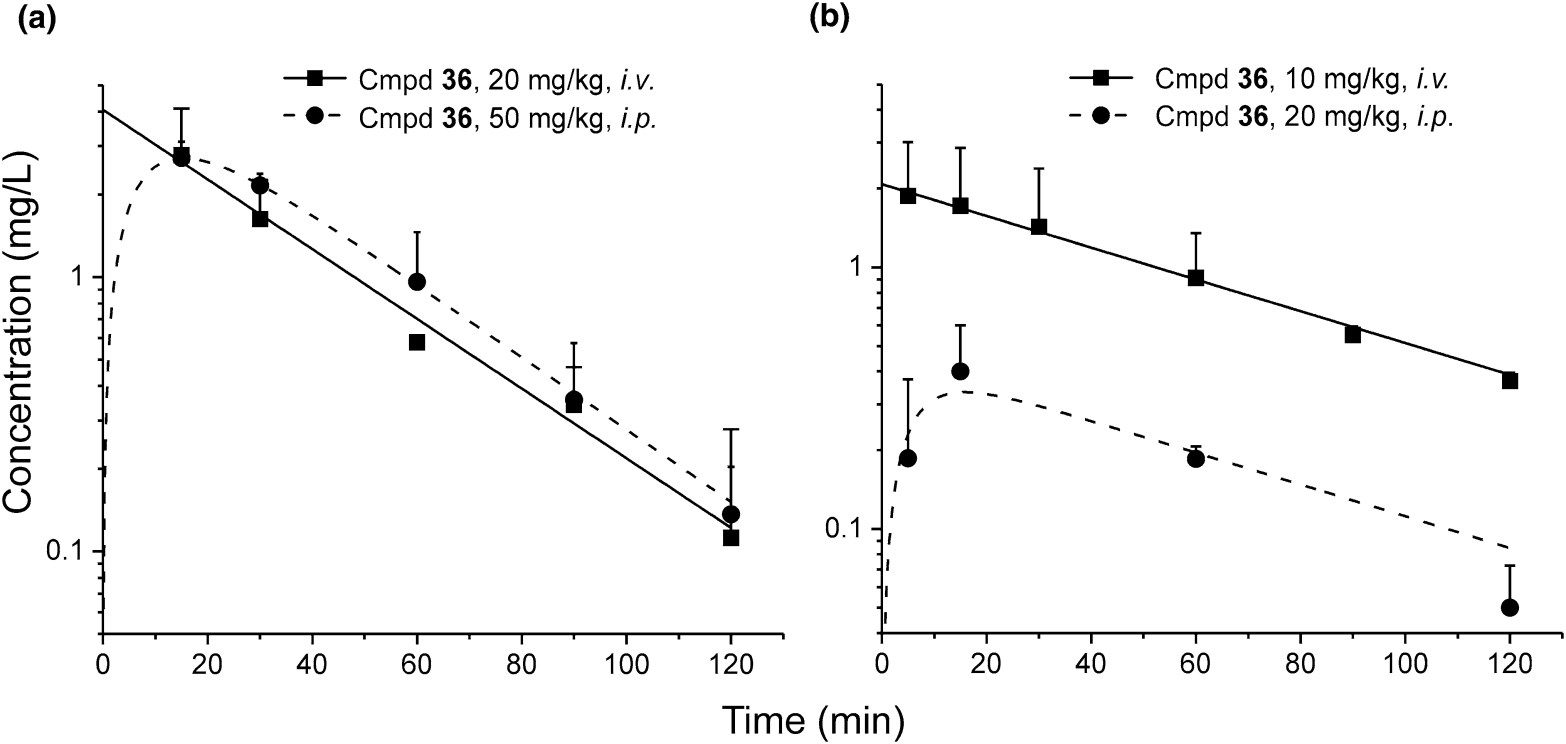
Observed (symbols) and pharmacokinetic model predicted (lines) compound **36** concentrations following iv (20 mg/kg) and ip (50 mg/kg) administration to male Wistar rats (**a**) and iv (10 mg/kg) or ip (20 mg/kg) administration to female Lewis rats (**b**) (*n* = 4)

The moderate activity of compound **36** observed in the CIA model, despite its strong anti-TNF-α activity [21], prompted us to compare its pharmacokinetic properties in both strains of rats used in this study. The results of pharmacokinetic analyses performed in both male Wistar and female Lewis rats after the administration of compound **36** via two different routes are illustrated in Fig. 3.

The pharmacokinetic parameters of compound **36** estimated in Wistar and Lewis rats using nonlinear regression were as follows: absorption rate constant—0.103 and 0.172 min^–1^, volume of distribution—4.53 and 4.81 L/kg, and elimination rate constant—0.033 and 0.014 min^–1^, respectively. The calculated elimination half-lives differed considerably in the two groups of rats (21 min in Wistar and 49.5 min in Lewis rats). The longer elimination half-life of compound **36** observed in Lewis rats may be related to its prolonged absorption from the peritoneal cavity, a phenomenon called flip-flop kinetics. The absolute bioavailability (F_a_) of compound **36** following ip administration in male Wistar rats was satisfactory (0.42), but it was considerably lower in the case of female Lewis rats (0.11).

## Discussion

Although numerous drugs are used in pain pharmacotherapy, their efficacy in the treatment of some types of pain is insufficient. Due to the rapid development of tolerance to analgesic activity, side effects, and drug dependence, researchers are searching for new medicines, focusing on mechanisms of activity other than those previously described, which may not only contribute to achieving more effective analgesia but also improving the safety of pain pharmacotherapy. In recent years, various phenomena and mechanisms responsible for nociception processes have been identified, which allows tailoring therapies based on the mechanism behind pain development.

Nowadays, studies conducted in research centers worldwide searching for new analgesic and/or anti-inflammatory active compounds mainly target, among others, PDEs. The PDE targets mainly analyzed are PDE4B, an isoenzyme that is expressed in inflammatory cells and whose inhibition plays a key role in anti-inflammatory effect, and PDE7A another cAMP-specific isoenzyme. Another target of interest is the TRPA1 ion channel, and more specifically, the main receptor of ion channels of the nociceptive system, which can be a critical factor in the development of pain, inflammation (e.g., inflammatory diseases of the respiratory tract, including asthma), and NP. Antagonists of these channels have been shown to reduce the infiltration of leukocytes and the levels of inflammatory cytokines [21]. Previously, it was found that TRPA1 functions as a sensor of oxidative and nitrosative stress by-products generated under various pathophysiological conditions in both the periphery and in the spinal cord. Recent evidence indicates that TRPA1 is also involved in the transition from acute to chronic pain. Since reactive oxygen species participate in the pathogenesis of pain and inflammation, TRPA1 channel antagonists are expected to exhibit antinociceptive, antiallodynic, and anti-inflammatory effects under various inflammatory conditions. Among numerous compounds identified as TRPA1 antagonists, only some (e.g. xanthine based acetamides including HC-030031) have shown the abovementioned effects in animal models of acute pain, inflammatory pain, and NP [9, 10, 12, 13].

The present study aimed to determine the pharmacological activity of two selected purine-2,6-dione-based TRPA1 channel antagonists and/or PDE4B/7A inhibitors—compounds **17** and **31** in comparison to **36**. The potential analgesic and anti-inflammatory/antiedematous activities of these compounds were evaluated in animal models of tonic pain and NP (by formalin and oxaliplatin-induced NP tests in mice) and inflammation (by carrageenan-induced edema test in rats).

The formalin test was carried out in a mouse model of chronic pain induced by the administration of 5% formalin solution [22]. The first phase of the test involves the stimulation of nociceptors and the development of neurogenic inflammation [22], and the second phase involves peripheral inflammation and central sensitization of pain. Since this phase reflects the activation of inflammatory processes, the compounds active in this phase also display an anti-inflammatory effect [22]. This model of pain may be useful to distinguish between the central and peripheral mechanisms of the analgesic action of newly designed compounds. Centrally acting compounds may be active in both phases of the test, while nonsteroidal anti-inflammatory drugs and glucocorticoids inhibit inflammation and pain only in the late phase.

Interestingly, all the compounds tested in the study were active in the second (inflammatory) phase of the formalin test, which indicates their peripheral mechanism of action. Compounds **17** and **31** showed a stronger antinociceptive and anti-inflammatory effect compared to the reference compound HC-030031 (Table 2). Compounds that have been found to show efficacy in the formalin test are TRPA1 channel antagonists and/or PDE4B/7A inhibitors. The most potent antinociceptive activity in the formalin test was exhibited by compound **36**, and its effect was tenfold higher than that of HC-030031 (ED_50_ = 3.4 mg/kg). The compound was also identified as the most potent PDE4B/7A inhibitor but a weaker TRPA1 channel antagonist in comparison to other tested compounds. The comparison of the ED_50_ values determined for compounds **17** and **31** in the formalin test indicated that both these compounds showed three- to fivefold higher antinociceptive activity than the reference compound HC-030031.

The results obtained for compounds **17** and **31** (and HC-030031 as a reference) indicate the important role of TRPA1 in pain development. It is well known that spinal TRPA1 channels contribute to the central sensitization of pain in rats [32]. Within the spinal dorsal horn, TRPA1 channels regulate the transmission of pain to spinal interneurons, and thus pain hypersensitivity. It has been shown that intrathecally administered Chembridge-5861528, a TRPA1 antagonist, suppressed formalin-induced secondary mechanical hypersensitivity, which highlights the involvement of spinal TRPA1 channels in secondary (central) pain hypersensitivity under various pain conditions [32]. These findings are in line with those of the present study. They also confirmed that the participation of TRPA1 channels in the inflammatory processes is a delayed effect that occurs over time. The distinct activity of compound **36** observed in the formalin test in the present study also indirectly suggests that distinct inflammation-related mechanisms and pronociceptive mediators are involved in the second phase (central sensitization and inflammation) as well as in the first phase of the formalin test (neurogenic inflammation). In the early (neurogenic) phase of the test, only compound **36** showed statistically significant antinociceptive activity, which indicates its central mechanism of action. The first phase of the formalin test may be a result of the direct and rapid activation of sensory C-fibers by peripheral stimuli (serotonin, bradykinin, substance P) and the development of neurogenic inflammation. In addition, our results showed that PDE inhibition might enhance the anti-inflammatory effect resulting from TRPA1 inhibition in the second phase of the formalin test, as observed for compound **36**. Recent studies have reported that PDEs are expressed in the dorsal horn of the spinal cord, demonstrating their potential contribution to pain processing, particularly inflammatory pain [33]. Moreover, the results of the present study suggest that PDE4B/7A inhibition might also enhance antinociceptive and anti-inflammatory responses in animals.

The study evaluated the effect of compounds **17** and **31** on NP because TRPA1 channel antagonists and PDE inhibitors are known to be effective against this type of pain [9, 10, 13, 14, 21]. Compound **17** was identified to be the most potent TRPA1 inhibitor, while compound **31** and also reference **36** were the PDE4B and PDE7A inhibitors, respectively.

In the von Frey test, compounds **17** and **31** showed promising antiallodynic profiles attenuating tactile allodynia in both phases of oxaliplatin-induced neuropathy. An overall effect of treatment was observed for these two compounds in oxaliplatin-treated mice in the cold plate test. However, compound **36** displayed a significant antiallodynic effect only at a dose of 10 mg/kg in the early phase of oxaliplatin-induced peripheral neuropathy. Because TRPA1 channel antagonists and/or PDE4B/7A inhibitors have shown efficacy in animal models of acute NP, they can be assumed to be effective against NP [9, 10, 13, 14, 21]. PDE7 inhibitors have been suggested to provide potential therapeutic benefits in the treatment of NP [14], and their effect on NP has been demonstrated in many animal studies [34]. This finding is confirmed by the present study because compounds **36** and **31** were identified as the most potent PDE7A inhibitors.

The carrageenan-induced edema test demonstrated that compound **31** significantly decreased the volume of edema induced by carrageenan injection into the hind paw of rats. Compound **31** statistically significantly reduced edema formation at 1 and 2 h of the experiment, by 62.0 and 48.3%, respectively. Compound **36** showed anti-inflammatory activity at 1 and 2 h of the experiment, inhibiting edema formation by 48.0 and 79.8%, respectively. The anti-inflammatory (antiedematous) activity of the tested compounds was higher than that of the reference compound HC030031, which statistically significantly inhibited edema development at 2 and 3 h of the experiment by 48.2 and 48.3%, respectively, at a dose of 100 mg/kg [21]. All the compounds examined in the carrageenan-induced edema model, showed a significant analgesic activity, but the compound **36** showed the strongest effect for which the analgesic activity was 63%.

Compound **36**, a potent dual PDE4/7 inhibitor, exerted strong anti-TNF-α activity in vivo, which was manifested by a significant reduction in the maximum plasma concentration of TNF-α by 90.2% in LPS-induced endotoxemia in rats [21]. On the other hand, compounds exhibiting only TRPA1 antagonistic properties did not show TNF-α-inhibiting potential [21]. Taking this into account as well as the fact that TNF-α is a key cytokine in the course of RA development, the activity of compound **36** was evaluated in rats with CIA.

The anti-inflammatory effect of compound **36** observed in rats with CIA in the present study seemed to be, at least partially, related to its TNF-α-inhibitory activity because TNF-α is a crucial mediator responsible for the progression of RA in humans and is also associated with the pathogenesis of CIA in rats [35]. Surprisingly, in this study, the antiedematous activity of compound **36** was found to be transient/temporary and dissipated since day 33 of the experiment. However, a similar phenomenon was observed by Redondo et al. for another PDE7 inhibitor, which exhibited only a temporary effect following its multiple administration in mice with MOG_35–55_-induced encephalomyelitis [36].

PDE4-selective inhibitors are clinically used to treat some immune-related disorders. For instance, roflumilast is used in the treatment of severe chronic obstructive pulmonary disease, and apremilast in psoriasis and psoriatic arthritis. Moreover, it has been reported both PDE4- and PDE7-selective, as well as PDE4/7 dual inhibitors, have the potential to ameliorate disease symptoms, decrease the levels of pro-inflammatory mediators, and increase the levels of anti-inflammatory cytokines in animal models of autoimmune disorders, including rats with CIA and mice with concanavalin-A-induced hepatitis or MOG_35–55_-induced encephalomyelitis [29, 37–39]. The abovementioned anti-inflammatory and immunoregulatory activities of PDE4/7 inhibitors (e.g., compound **36**) are mainly related to their ability to increase cAMP concentrations in immune cells, which subsequently activates the cAMP/PKA pathway regulating the expression of inflammatory mediators [40].

Because compound **36** showed moderate activity in the CIA model, their pharmacokinetic profile was analyzed. The obtained results indicated the low absolute bioavailability of compound **36** following its ip administration in female Lewis rats, suggesting that the blood concentrations of this compound might be insufficient to obtain a full therapeutic effect. Another possible explanation for the lower-than-expected activity of compound **36** in the CIA model could be the autoinduction of its metabolism during multiple dosing, leading to insufficient levels to achieve the desired pharmacological effect. The observed moderate effect might also be linked with some adaptive changes that occurred in immune cells, such as upregulated expression of different isoforms of PDE in response to elevated cAMP levels. Despite significant advances in the development of biologics for the treatment of RA, methotrexate (MTX) remains the gold standard for this disease. In the study of Liu et al. [28], MTX was administered subcutaneously at doses of 0.3 and 1.5 mg/kg on alternate days in Lewis rats with CIA. A reduction in paw area by 9.6 and 7.1% was observed in the group treated with the lower dose of MTX on days 27 and 31 of the experiment. Moreover, the observed therapeutic effect was sustained until the last day of the experiment (day 43), with a reduction in paw swelling by 11.1% compared to the vehicle-treated group. In contrast, no improvement was observed in the group treated with the higher dose of MTX, but considerable toxicity was noted, which was manifested by increased mortality, bleeding, diarrhea, and weight loss.

The results of our study indicate that compound **36** exhibited desired therapeutic effect and improved safety profile compared to MTX, since no toxic effects, which were observed during MTX treatment, were noted during the 20 days multiple dosing of this compound in arthritic rats. The anti-inflammatory activity of compound **36** observed in rats with CIA could be probably potentiated by increasing its dose or changing the administration route to achieve higher concentrations at the site of action.

Our study showed that 8-alkoxypurine-2,6-diones may represent a new class of compounds with analgesic and anti-inflammatory activities. Compound **36** was identified as the most promising multifunctional TRPA1 antagonist and a PDE4B/7A dual inhibitor. It inhibited both cAMP-specific PDE isoenzymes resulting in an antiarthritic effect in the CIA model. Moreover, compound **36** exhibited potent anti-inflammatory and analgesic activities in animal models of pain and inflammation (the formalin test in mice and the carrageenan-induced edema test in rats) and displayed significant antiallodynic properties in the early phase of chemotherapy-induced peripheral neuropathy in mice. The moderate activity of compound **36** observed in the CIA model prompted us to evaluate its pharmacokinetic profile in both strains of rats used in this study. The absolute bioavailability (F_a_) of compound **36** was satisfactory in male Wistar rats, but it was four-fold lower in the case of female Lewis rats.

## Conclusion

The findings of this study confirmed that administration of a TRPA1 antagonist with potent PDE4B/7A inhibitory activity may induce an enhanced analgesic effect, at least against certain types of pain, compared to that of pure TRPA1 antagonists.

The proposed pain treatment approach, which is based on the concomitant blocking of the TRPA1 channel and PDE4B/7A, appears to be an interesting research direction for the future search for novel analgesics. Importantly, some of the biologically active structures that can inhibit both TRPA1 and PDE4B/7A may increase the pharmacological effect of drugs currently used in the treatment of NP (pregabalin). This may allow achieving desired therapeutic effect at lower doses and improve the efficacy and safety of pain pharmacotherapy.

## Supplementary Information

Below is the link to the electronic supplementary material.

Supplementary file1 (XLSX 60 KB)

Supplementary file2 (XLSX 13 KB)

## Data Availability

The data supporting the findings of this study are available in Supplementary materials.
